# Infrared light therapy relieves TLR-4 dependent hyper-inflammation of the type induced by COVID-19

**DOI:** 10.1080/19420889.2021.1965718

**Published:** 2021-09-15

**Authors:** Blanche Aguida, Marootpong Pooam, Margaret Ahmad, Nathalie Jourdan

**Affiliations:** a Cnrs, Ibps, Sorbonne Université, Paris, France; b Department of Biology, Faculty of Science, Naresuan University, Phitsanulok, Thailand; c Department of Biology, Xavier University, Cincinnati, Ohio, USA

**Keywords:** Photobiomodulation therapy, reactive oxygen species, inflammation, Covid19, infrared therapy, cytokine storms

## Abstract

The leading cause of mortality from COVID-19 infection is respiratory distress due to an exaggerated host immune response, resulting in hyper-inflammation and ensuing cytokine storms in the lungs. Current drug-based therapies are of limited efficacy, costly, and have potential negative side effects. By contrast, photobiomodulation therapy, which involves periodic brief exposure to red or infrared light, is a noninvasive, safe, and affordable method that is currently being used to treat a wide range of diseases with underlying inflammatory conditions. Here, we show that exposure to two 10-min, high-intensity periods per day of infrared light causes a marked reduction in the TLR-4 dependent inflammatory response pathway, which has been implicated in the onset of cytokine storms in COVID-19 patients. Infrared light exposure resulted in a significant decline in NFkB and AP1 activity as measured by the reporter gene assay; decreased expression of inflammatory marker genes IL-6, IL-8, TNF-alpha, INF-alpha, and INF-beta as determined by qPCR gene expression assay; and an 80% decline in secreted cytokine IL6 as measured by ELISA assay in cultured human cells. All of these changes occurred after only 48 hours of treatment. We suggest that an underlying cellular mechanism involving modulation of ROS may downregulate the host immune response after Infrared Light exposure, leading to decrease in inflammation. We further discuss technical considerations involving light sources and exposure conditions to put these observations into potential clinical use to treat COVID-19 induced mortality.

## 1 Introduction

Coronaviruses are pleiomorphic RNA viruses, which have caused multiple epidemics in the past 20 years, including SARS (Severe Acute Respiratory Syndrome), MERS (Middle East Acute Respiratory Syndrome), and most recently the global SARS-COVID-19 pandemic [1,2]. All variants are characterized by lack of an effective antiviral treatment and, due to their mutation rates, the long-term efficacy of newly developed vaccines is not assured. Hence, there is an ongoing need to develop efficient therapeutic interventions against the induced severe respiratory distress, which is a leading cause of death [3].

The excessive, uncontrolled inflammatory response leading to acute lung injury and morbidity is a known consequence of coronavirus infections [3–5]. In particular, the activation of the TLR4 receptor, a pathogen pattern recognition receptor of the innate immune system, was found to play a central role in the onset of hyper-inflammation and cytokine storms [6]. The mechanism of action of TLR-4 is well understood at the molecular level and its deregulation leads to excessive production of pro-inflammatory chemokines and cytokines including TNF-alpha, Il-1Beta, and Il-6, and as a consequence it is toxic in a number of viral diseases [7,8].

The TLR4 signaling pathway has been directly implicated in cytokine storms induced by SARS-CoV-1 [9], as well as in SARS-CoV-2 infection [10]. Indeed, after first being predicted by immunoinformatic approaches [11,12], a direct interaction between SRAS-CoV-2 spike protein and TLR 4 was confirmed experimentally at the molecular and cellular levels [13–15]. Moreover, purified spike protein induces expression of inflammatory cytokines as robustly as does LPS in a human monocyte cell line [13]. By contrast, again in human monocyte cell lines, reasorvid (an inhibitor of TLR4) significantly blocks induction of inflammatory cytokines by spike protein and LPS. This clearly indicates that SARS-CoV-2 spike protein interacts with and activates the TLR4 signaling pathway by the identical mechanism and leads to the same high-level production of pro-inflammatory cytokines as does LPS [13]. The TLR-4 receptor is present in lung alveolar cells, as well as alveolar macrophages and lung fibroblasts [16]. Furthermore, the TLR-4 dependent hyperactive response has been implicated in acute lung injury, whereas downregulation of the TLR4 receptor reportedly attenuates injury. In animal studies, TLR-4 receptor mutant mice showed enhanced protection from acute respiratory distress induced by SARS-CoV-1 infection [9]. Other studies also showed protection from acute lung inflammation due to IAV, EBOV, and DENV viral infection as a result of TLR4 receptor antagonistic drugs [6].

In consequence, a therapy that targets the TLR4-dependent inflammatory response could in principle be effective against the respiratory distress and cytokine storms caused by SARS-CoV-2 in COVID-19. Treatments that inhibit downstream selective cytokine signaling have been used in patients with COVID-19. Anti-human IL-6 receptor monoclonal antibody (tocilizumab and sarilumab) [17–19] as well as IL-1 receptor antagonist (anakinra) [18] provided a significant survival advantage but were less effective against late-stage COVID-19 [18]. Moreover, as IL1 and IL6 are not the only cytokines responsible for hyperinflammation, a treatment that targets a step upstream of cytokine synthesis in the inflammatory cascade would probably be more effective against severe ARDS.

As a possible solution, a noninvasive, illumination-based therapy known as photobiomodulation therapy has been suggested as a treatment for COVID-19 [20,21]. Photobiomodulation is a proven treatment against various conditions involving resolution of underlying inflammation that include Achilles tendinopathy [22]. Alopecia Areata [23,24], psoriasis [25,26], thyroiditis [27], and arthritis [28,29]. In experimental animal models that reproduce allergic lung inflammation [30,31] or acute lung injury induced by gut ischemia and reperfusion [32,33] or sepsis [34], laser irradiation has produced anti-inflammatory effects related to the downregulation of leukocyte attractant chemokines [30–33], and by attenuation of ROS generation [30,31,33].

Photobiomodulation therapy involves short periods of illumination with red and/or infrared light, given repetitively over several days [28]. The underlying principle is that these short illuminations trigger transient oxidative bursts in the mitochondria of the exposed tissue, leading to regenerative and/or anti-inflammatory effects [28,35]. The advantage of such treatments is that they can be targeted directly to the affected organ or body part, unlike drugs, which must be ingested and then will target all cells equally. Importantly, there are no known harmful side effects as such illuminations do not cause tissue damage. In further support of photobiomodulation therapy in treating COVID-19, it was recently shown that infrared light exposure has been effective in reducing the inflammatory response in a variant of human HEK293 cell cultures [36], and a number of case reports involving application of photobiomodulation therapy on COVID-19 patients [37–39].

In the present study, we perform a detailed analysis of the effect of infrared light on the inflammatory response in commercially available HEK-Blue^TM^ hTLR4 human cell cultures (see Methods). These cells have been engineered to express the human TLR4 receptor and MD2/C14 co factors and contain all needed downstream signaling intermediates leading to activation of NF-kB and AP-1, inducing transcription of pro-inflammatory cytokines. By using bacterial lipopolysaccharide (LPS) or other TLR4 agonists, transcription of proinflammatory cytokines can be detected by colorimetric quantification of secreted embryonic alkaline phosphatase (SEAP), a reporter gene placed under the control of NFkB and AP1-binding sites. HEK-Blue^TM^ hTLR4 cell lines that fully replicate the TLR4-dependent inflammatory pathway have in fact been widely used to study efficacy of pharmaceutical remedies for inflammation [40–49]. In the present work, we have quantitated the effects of infrared light on inflammation and established the optimum light intensities and durations of exposure, as well as the effectiveness of different types of lights.

The goal was to ascertain whether photobiomodulation could form the basis for a viable therapeutic solution to COVID-19 induced respiratory distress, and to establish the optimal treatment dose.

## 2 Materials and methods

### 2.1 Cell cultures and growth conditions

Cell culture conditions are taken from reference [49] and were performed as follows: Human embryonic kidney HEK293 cell lines https://www.invivogen.com/hek-blue-htlr4, stably expressing human TLR4 (InvivoGen, San Diego, CA, USA), were used for all experiments. HEK-Blue^TM^ hTLR4 cells express an alkaline phosphatase (SEAP) reporter gene regulated by NF-κB and AP1 transcription factors. The quantification of cell-infection was measured by assaying alkaline phosphatase activity in cell culture medium containing colorimetric enzyme substrates.

Cells were cultured in DMEM high glucose (Dulbecco′s Modified Eagle Medium (DMEM), Sigma, St. Louis, MO) containing 4500 mg/l of glucose, 10% (v/v) heat-inactivated fetal bovine serum (Gibco, Dublin, Ireland) and 1x HEK-Blue Selection solution (InvivoGen, San Diego, CA, USA) and grown at 37°C under a humidified atmosphere at 5% CO_2_ in a dedicated incubator (MCO-18AC, Panasonic Biomedical, Leicestershire, UK).

Cells were first amplified in 75 ml culture flasks and sub-cultured every 72 hours. In experimental trials, HEK-TLR4 cells were seeded from a single stock culture flask at a density of 2 × 10^4^ cells per well in 96-well plates. Inflammatory response was stimulated at seeding by incubation with bacterial lipopolysaccharide (LPS) dissolved in Phosphate Buffered Saline (PBS) (Sigma, MO, USA). A final concentration of 100 ng/ml LPS was used for all tests. Negative control cultures were obtained by adding inert physiological saline (PBS) at the same volume as the LPS substrate to the control culture medium. After LPS addition, cell cultures were incubated for a further 16 hours before transfer to the relevant exposure conditions (Infrared light). All cultures were grown in parallel from the same cell stock culture and under identical conditions.

### 2.2 Light sources

Infrared exposure was achieved using several different illumination sources as indicated. For the LED sources, we have used interchangeably a pre-mounted 7-LED high output LED array (720 nm) (www.luxeonstar.com) from Quadica inc., Alberta, Canada; high output LED infrared floodlights or bulbs ref# N300; #IR720nm AF; #IR720nm AB (www.synlyte.com) from Synlyte SAS, Massy Palaiseau, France. The incandescent light source (IC light) was the Philips PAR38E 150 W incandescent bulb (https://www.lampesdirect.fr/philips-par38-ir-150w-e27-230v-rouge-8711500128874). The wavelength range of all LED light sources is between 720 and 750 nm. All infra red LED light sources worked equally well for treatment and were used interchangeably.

### 2.3 Light exposure conditions

The LED or incandescent light source was placed 20 cm above the 96-well culture plate to create a uniform beam for illumination of the culture plates. Infrared light intensity from the different light sources was adjusted by placing layers of paper filters between the lamp and the sample. Light intensity at the position of the cell culture dish was detected by a Quantum light meter (LI-185B, Li-Cor, Lincoln, NE, USA) with a pyranometer probe (Li-Cor, Lincoln, NE, USA).

Activation of the LEDs during cell culture was controlled by a custom-built automated programmable switch as described previously (Pooam et al., 2019). Briefly, the switch was created using a 4-channel 5 V power relay board using GPIO pins of a Raspberry Pi 3B Light Starter kit to power on infrared LED illumination. The infrared sequence was programmed to switch on for 10 min every 12 h over a total time of 48 h. The control condition was performed in an identical manner (inflammation was induced with 100 ng/ml LPS), except that cells were cultured in the incubator without infrared illumination.

### 2.4 Alkaline phosphatase assay for monitoring inflammation

The inflammatory response of HEK-TLR4 cells was measured by determining the enzyme activity of the secreted alkaline phosphatase (SEAP) reporter gene, which was normalized to the total concentration of cells per well. SEAP enzyme activity was assayed at the end of the 48-h growth period by removing the equivalent of 7 µl of cell-free supernatants from each of the five duplicate wells subjected to the treatment condition. The culture media samples were then mixed with 180 µl of QUANTI-Blue^TM^ detection solution (Invivogen), which contains the AP colorimetric substrate, and incubated in accordance with manufacturer’s specifications at 37°C, 5% CO2 for 20 min in a fresh 96-well plate. Alkaline phosphatase activity was measured as the absorbance of the detection solution at 620 nm using an Epoch microplate reader (BioTek, Winooski, Vermont, USA). Values from five duplicate wells were averaged to obtain a single experimental data point.

In order to detect possible differential cell growth effects resulting from these treatments, the HEK-TLR4 cells were also measured for total protein concentration in each well after the treatment period, using the DC Protein Assay kit (Bio‐Rad Laboratories, Mississauga, ON, Canada). Briefly, the culture medium was removed from each of the five duplicate wells subjected to experimental conditions. 30 µl of cell lysis buffer (25 mM Tris-HCl pH 7.4, 150 mM NaCl, 1% NP-40, 1 mM EDTA, 5% glycerol) was added to the cells inside the culture wells and incubated for 1 h at 4°C to induce cell lysis and achieve protein solubilization. 15 µl of lysate was then transferred into a fresh 96-well plate and mixed with the DC protein assay reagents as recommended by the manufacturer. The levels of total proteins were measured by absorbance at 750 nm by an Epoch microplate reader (BioTek). The absorbance value of QUANTI-Blue^TM^ Solution (OD620), representing secreted alkaline phosphatase activity, was subsequently normalized to the total protein concentration (OD750) and presented as a ratio (OD620/OD750).

A background level of alkaline phosphatase secretion was observed in cell cultures that had not been exposed to LPS after the 48-h incubation period, which did not respond to anti-inflammatory treatments. This background SEAP value was subtracted from the values obtained from the LPS – stimulated cell cultures to obtain the TLR-4 dependent component of the inflammatory response. The effect of treatments is expressed as the percentage of inflammation achieved after LPS induction in IR treated groups as compared to the SEAP secretion response of untreated control cells that had received LPS stimulation.

### 2.5 Quantitative RT-PCR analysis of altered gene expression

The qPCR analysis was performed as described [36]. After exposure to each treatment condition, the total RNA was extracted from HEK-TLR4 cells by Total RNA Miniprep Kit (New England Biolabs), according to the manufacturer’s instructions. cDNA was prepared from 1 µg total RNA using ProtoScript® II First Strand cDNA Synthesis Kit (New England Biolabs). Quantitative RT-PCR was performed using Luna qPCR master mix (New England Biolabs). The GADPH gene was used as the reference gene. Quantitative RT-PCR was performed by Mastercycler® RealPlex2 (Eppendorf). Three biological replicates were performed for each gene (N = 3). Data analysis to represent the relative expression level of genes of interest was performed as previously described [27]. Primers (primer from [50] and purchased from Eurogentec) used for gene expression analysis are described in Table 1.

**Table 1.** List of primers used in the current study

Species	Genes	Forward Primer 5ʹ-3’	Reverse Primer 5ʹ-3’
**Human**	**IL-6**	GGCTGCAGGACATGACAACT	ATCTGAGGTGCCCATGCTAC
	**IL-8**	CCACCGGAAGGAACCATCTC	GGGGTGGAAAGGTTTGGAGT
	**TNFα**	CAAGGACAGCAGAGGACCAG	TGGCGTCTGAAGGTTGTTTT
	**IFNα**	AGAATCACTCTCTATCTGAAAGAGAAG	TCATGATTTCTGCTCTGACAACCT
	**IFNβ**	CGCCGCATTGACCATCTA	GACATTAGCCAGGAGGTTCT
	**GAPDH**	ATTCCACCCATGGCAAATTC	CGCTCCTGGAAGATGGTGAT

### 2.6 ELISA assay for IL – 6

HEK-TLR4 cells were seeded at a density of 1.300.000 cells in 22.1 mm^2^ plates and stimulated with 100 ng/mL of LPS (TLR4 ligand), causing hyper – inflammation. Cells were cultured at 37°C and 5% CO2. After 24 h, cells were illuminated 10 min with infrared light every 12 h for 48 h. Infrared LEDs generated light at an intensity of 6 W/m^2^. Photon fluence of infrared intensity for the experiment was detected by a quantum light meter (LI-185B, Li-Cor, Lincoln, NE, USA) with a pyranometer probe (Li-Cor, Lincoln, NE, USA). During the stimulation, LEDs were placed 20 cm above the culture plates. Stimulation was for 10 min every 12 h, for a total of 48 h. On Day 5, 12 h after the final stimulation with infrared light, the cell-free supernatant of both treatment (Infrared Light exposed) and control (un illuminated) cells was replaced with a fresh medium containing 100 ng/mL LPS. Cells were then incubated in a fresh medium at 37°C and 5% CO2 for an additional 6 hours. The cell-free supernatants were then harvested, and the cytokine IL-6 secreted into the media during the final 6 hours (Day 5) of the experiment was measured using commercially available ELISA kits (https://www.enzolifesciences.com/ENZ-KIT178/il-6-human-high-sensitivity-elisa-kit). The kit is specific for human IL-6 and was used according to the manufacturers’ instructions. This kit has a high sensitivity (0.057 pg/mL) for IL-6 quantification. The control condition was performed in an identical manner (with 100 ng/ml LPS), but without exposure to infrared.

### 2.7 Statistical analysis

All data were analyzed by using GraphPad Prism version 7.4.2 for Mac (GraphPad Software, La Jolla California, USA). Data were analyzed for normality with the Shapiro–Wilk test. The results will be expressed as mean ± standard error of the mean (SEM). The differences between treated and control conditions for each gene were compared using one-way ANOVA followed by Tukey’s multiple comparisons test. Comparisons were of the Exposed (to a given infrared treatment with LPS stimulation) to the Control (Dark with LPS stimulation) sample grown at the same time from the same cell stock. Differences were considered statistically significant with a *p*-value <0.05 (*), <0.01 (**), <0.001 (***), <0.0001 (****).

## 3. Results

### 3.1 Colorimetric assay to detect therapeutic effect of infrared light on inflammation

**Figure 1.**
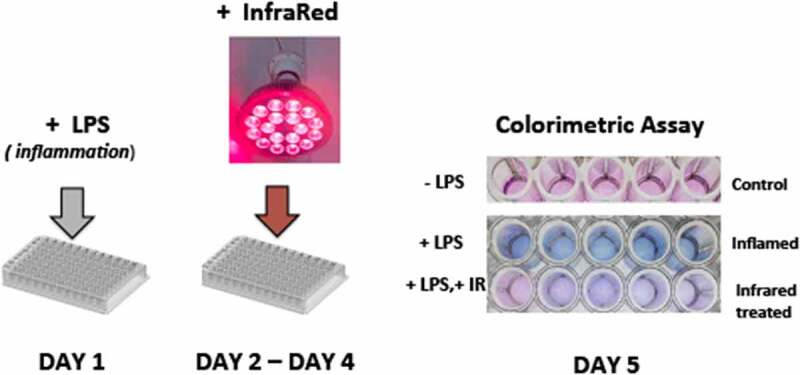
Inflammatory response to 720 nm Infrared Light in HEK293 cell cultures. DAY 1: cells were seeded into 96 well culture plates and exposed to LPS (bacterial lipopolysaccharide). Five duplicate wells were seeded per sample condition. DAY 2 – DAY 4: Cell cultures were exposed to 10 m of infrared LED light, 6 W/m^2^, applied once every 12 hours. DAY 5: Colorimetric assay of cell culture medium performed as in METHODS; blue coloration occurs as a consequence of the inflammatory response. Infrared Light exposure visibly decreases the inflammatory response. Results shown are from a single representative experiment

The HEK – TLR4 cell lines used in this study incorporate the TLR4, MD-2 and CD14 co-receptor genes and can be induced to undergo a TLR4 – induced inflammatory response by addition of bacterial lipopolysaccharide (LPS) to the cell culture medium. In addition, this cell line is stably transformed with a secreted reporter gene (SEAP – secreted embryonic alkaline phosphatase) under the control of a promoter that can be activated by inflammation response regulators AP1 and NFk1 transcription factors. In this way, the progress of the inflammatory response can be monitored by a simple colorimetric assay (Figure 1). To test the effect of exposure to Infrared light on these cell cultures, we first seeded the cells on culture plates and added LPS as described in Methods to induce the inflammatory response. After 24 hours, cell cultures were exposed to Infrared (720 nm) LED light at 6 W/m2 for 10 minutes in the incubator. The infrared illumination treatment was continued once every 12 hours for 48 hours (a total of 4 Infrared treatments in all). Twelve hours after the final illumination treatment, the cell culture media was analyzed for the secretion of SEAP, as determined by a blue color in the colorimetric assay (methods). Under these conditions, there was a visible decrease in blue color in cell cultures that had been exposed to Infrared Light as compared to control cell cultures (Figure 1). This result is indicative of approximately 50% decline in inflammatory response as a result of treatment.

### 3.2 Therapeutic effect of infrared light as a function of dosage

The wavelength of 720 nm – 750 nm infrared light was initially chosen because it is one of the most effective in penetrating at depth into living tissue, while at the same time, it does not generate heat. In a preliminary study, it was determined that exposure intensities of 720 nm light ranging from 2 to 6 W/m^2^ at the position of the cell sample were equally effective in treating inflammation.

**Figure 2.**
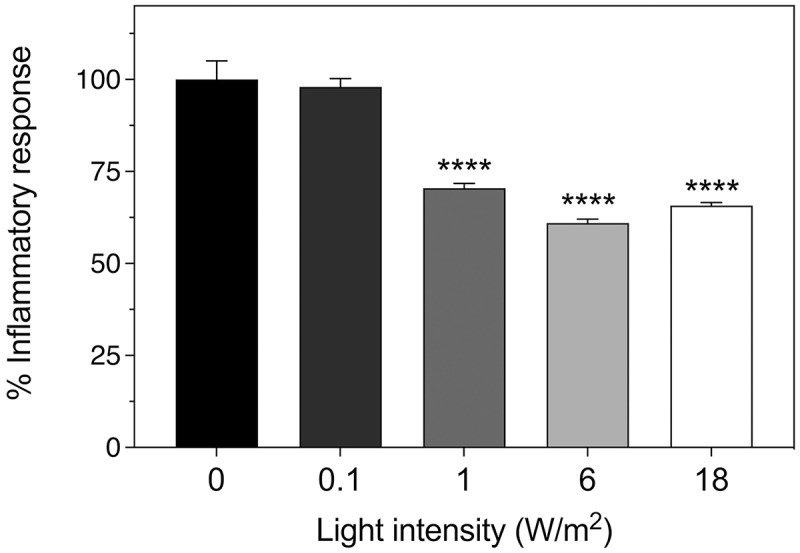
**Light intensity dependence of anti-inflammatory response of HEK cells**. The inflammatory response was induced in cell cultures by incubation with 100 ng/ml LPS and followed by exposure to 720 nm infrared illumination at the indicated intensities as described in methods. The control condition represents inflammatory response of HEK-TLR4 cells exposed to 100 ng/ml LPS with no exposure to infrared light. The decrease in the inflammatory response after exposure to IR light is expressed as a percentage of the control. Data represent the mean ± SE of four independent experiments (N = 4). The asterisks indicate significance level of the differences: ***p-value < 0.001

Here, we extend this range to include higher (18 W/m^2^) and much lower (0.1 W/m^2^) light intensities. The results show that there is no change between 6 and 18 W/m^2^, indicating that the former intensity is already at saturation for mitigating the inflammatory response. However, there was a 30% decline in anti-inflammatory effectiveness of the treatment at 1 W/m^2^ and negligible response at 0.1 W/m^2^ (Figure 2). Our results, therefore, define a therapeutic dose of 6 W/m^2^ intensity at the cellular level. Importantly, a greatly increased intensity (even 8-fold above the minimum effective treatment intensity of 2 W/m^2^) had no harmful effects on the cells and was still fully therapeutic. This means that only the minimum therapeutic dose must be calibrated, and that any excess above the minimum has no harmful effects.

**Figure 3.**
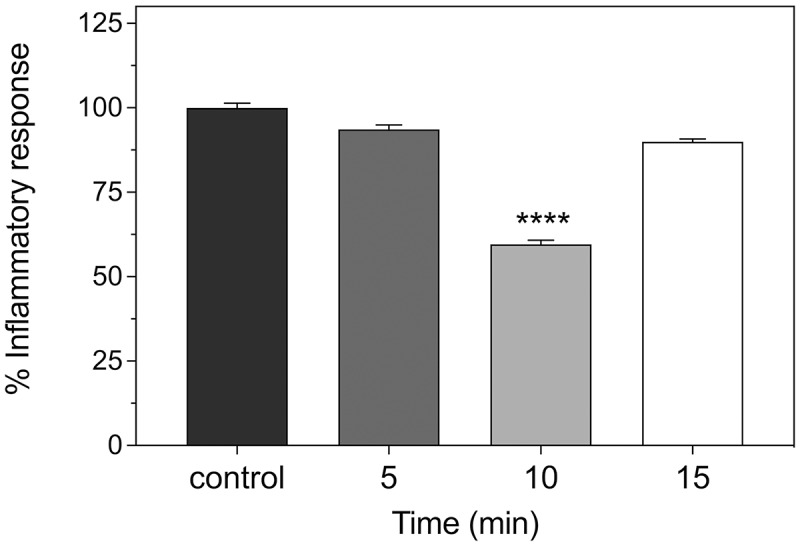
**Time dependence of anti-inflammatory response of HEK cells**. The inflammatory response was induced in HEK-TLR4 cell cultures by incubation with 100 ng/ml LPS and followed by exposure to 720 nm infrared illumination under the indicated conditions. Inflammatory response was determined by colorimetric assay (methods). The control condition represents the inflammatory response in the absence of Infrared exposure. Exposure to infrared was for 5, 10, and 15-min intervals, respectively. The intervals were repeated once every 12 h, over 48 h for a total of four Infrared exposure periods. The Inflammatory response was assayed by colorimetric assay at 12 h after the final Infrared treatment. Data represent the mean ± SE of three independent experiments (N = 3). The asterisks indicate significance level of the differences: ****p-value < 0.0001

A further parameter in determining the optimum therapeutic light dose involves the question of the time-dependence of irradiance. Traditionally, photobiomodulation therapy treatments are given over a range of times varying between 5 min and 1 h, once or twice a day [28]. In prior experiments, it was ascertained that twice daily irradiations were most effective in treating the inflammatory response [36]. However, the 10-min duration time was obtained empirically, and longer or shorter exposure times were not tested. Here, we have exposed the cell cultures to either 5, 10, or 15 min of infrared light exposure, twice daily, over a 48-h treatment period (methods), to determine the optimum conditions. The inflammatory response was measured by colorimetric assay at the end of the treatment, and compared to the untreated control cultures. Our results (Figure 3) determine that both 5- and 15-min exposure time show only moderate effects (less than 10% decrease in inflammatory markers) as compared to the optimum of 50% decrease at the 10-min exposure time. Therefore, the duration of the exposure is a critical parameter to induce an effective anti-inflammatory response.

### 3.3 Analysis of gene expression markers for inflammation

**Figure 4.**
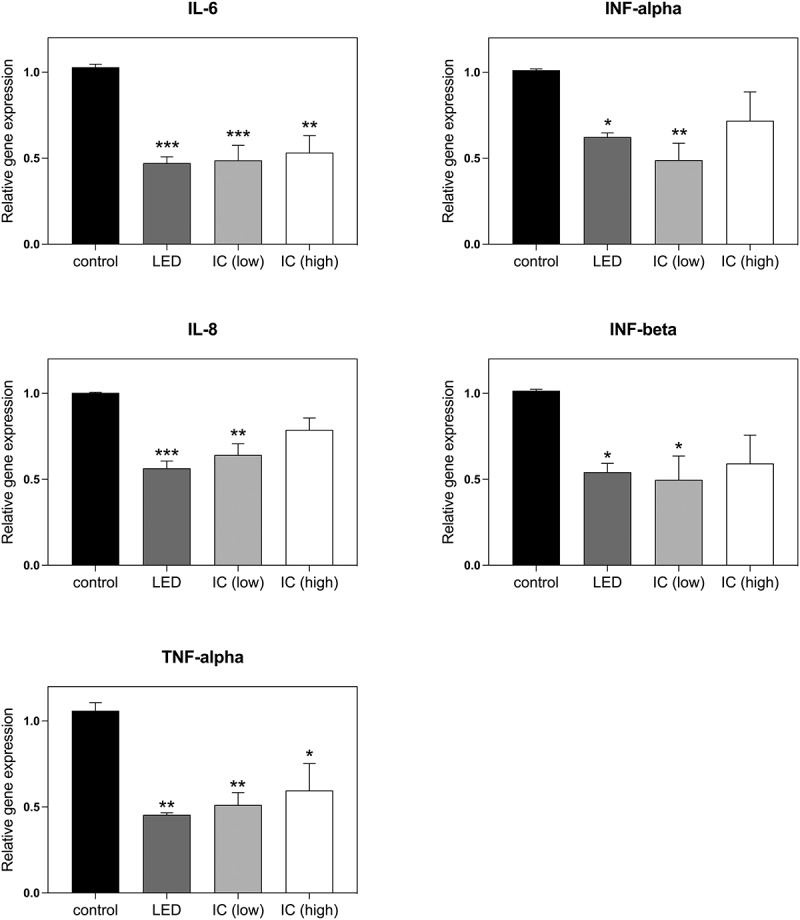
**Gene expression analysis of Effect of Infrared Light on Inflammatory markers**. Genes induced during the inflammatory response in HEK-TLR4 cell cultures including inflammatory cytokines (IL-6, IL-8) and transcription factors (TNF-α, INF-α, INF-β) were monitored by qPCR analysis subsequent to induction of the inflammatory response by LPS. The control condition represents the expression levels of cell cultures that had not been exposed to infrared light. In the other conditions, cultures were exposed to infrared light for 10 m every 12 h for 48 h as follows: LED: exposure to 6 W/m^2^ infrared 720 nm LED lights, IC (low): exposure to 6 W/m^2^ incandescent infrared light bulb (methods); IC (high): exposure to 46 W/m^2^ incandescent infrared light bulb (methods). Data are shown as mean ± SE of four independent experiments (N = 4). The asterisks indicate significance level of the differences: *p-value < 0.1; ** *p*-value < 0.01; *** *p*-value < 0.001

A number of cytokines are induced in the course of the TLR4-mediated hyper-inflammatory response. Among the most important are genes for the inflammatory cytokines Il-6, IL-8, TNFα; and interferons IFNα and IFNβ [7]. We have accordingly performed qPCR gene expression analysis to determine the response of these inflammatory marker genes to infrared illumination. The inflammatory response was induced in cell cultures by LPS, followed by exposure to 720 nm LED light source at an intensity of 6 W/m^2^ for 10 min at 12-h intervals (see methods). As an additional light source, we also tried an incandescent infrared light bulb whose emission wavelength range also includes 720 nm (see methods). At the end of the 48-h treatment period, cells were harvested and gene expression analysis performed. The results showed an approximately 50% reduction in gene expression of all of these markers in response to LED infrared exposure (Figure 4). Incandescent infrared light exposure was also effective at both low (6 W/m^2^) and high (46 W/m^2^) intensity. However, we subsequently determined that infrared bulbs cannot be used for treatment in human patients because the heat generated at the skin surface was at 72°C in the intensity range (over 1000 W/m^2^) required to penetrate the chest cavity [36] and therefore would cause serious burns.

### 3.4. ELISA detection of Il-6 cytokine secretion as a function of Infrared Light exposure

Some of the most damaging effects of the uncontrolled auto-inflammatory response unleashed by COVID-19 occurs as a result of excess cytokine secretion, leading to destruction of the alveolar tissue and respiratory distress. Cytokine 6 (IL – 6) in particular has been associated with COVID-19 infection. We therefore directly determined the effect of infrared exposure on the secretion of IL6 by HEK-TLR4 cells by performing an ELISA assay.

**Figure 5.**
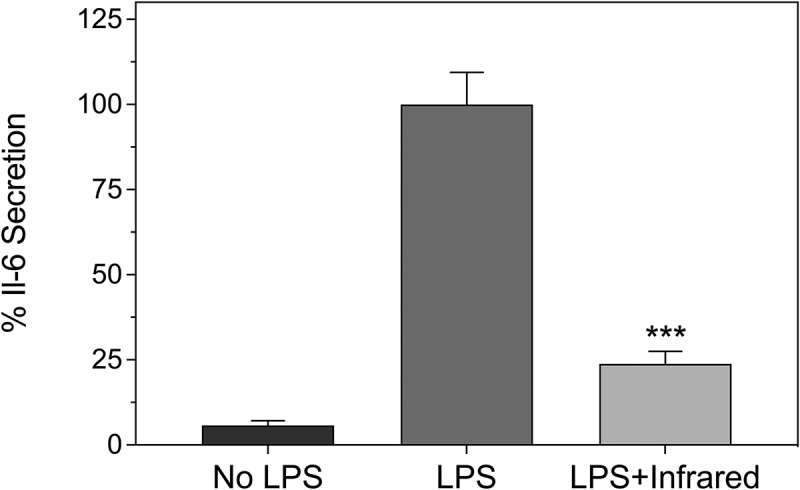
**Decline in IL-6 secretion in cell cultures as a result of infrared therapy**. HEK-TLR4 cell cultures were treated with or without 100 ng/ml LPS to induce inflammation as described (see Methods). The cell cultures were subsequently maintained in the dark (No LPS, LPS) or exposed to infrared light (10 m at 6 W/m2, once every 12 hours) for 48 h (LPS + Infrared). Secreted Il-6 was measured using an ELISA kit according to the manufacturer’s instructions (methods). Il-6 secretion is expressed relative to the LPS stimulated cell cultures (100%). Data are shown as mean ± SE of three experiments

The inflammatory response was induced in cell cultures by incubation with 100 ng/ml LPS and followed by exposure to 720 nm infrared illumination at 6 W/m2 for 10 min every 12 h as described in Methods. The control condition represents the inflammatory response of HEK-TLR4 cells exposed to 100 ng/ml LPS with no subsequent exposure to infrared light. After the last infrared exposure treatment (48 h), the cell culture media was replaced with fresh media also containing inflammatory LPS. The cells were allowed to grow for an additional 6 h before assaying for the presence of secreted cytokine 6 using an ELISA test kit (see methods). The results showed that, after only 2 days of infrared treatment, the level of Il-6 secretion was decreased by almost 80% relative to the untreated cell cultures (Figure 5). If a comparable decline in IL-6 secretion is also induced in patients, infrared treatment could be of real therapeutic benefit for late-stage COVID-19 patients.

## 4. Discussion

In this study, we have shown that infrared light exposure can significantly decrease inflammation caused by the TLR-4 dependent innate immune response in a model human cell culture system. As measured by colorimetric substrate, infrared light exposure caused a decrease in inflammation by 35%. Gene expression markers for inflammation were decreased by up to 50% after only 2 days of treatment, whereas IL-6 cytokine secretion was decreased by 75% by the end of the treatment period. Because the TLR-4 dependent signaling pathway becomes deregulated as a result of SARS viral infection, our results suggest that infrared therapy could provide a safe, affordable, and effective means of treating the advanced respiratory distress syndrome and cytokine storms that are a leading cause of death.

### 4.1 Relevance of findings in HEK – TLR4 cells to the treatment of COVID-19 patients

Deregulation of the TLR4 dependent immune pathway is characteristic of the human respiratory pathogenic beta-coronavirus subfamily (SARS/MERS), leading to so-called cytokine storms and secretion of toxic concentrations of IL-6 and other cytokines [6,45]. The TLR4 signaling pathway is highly conserved and occurs in both alveolar and resident lung macrophage cells, suggesting that a therapeutic mechanism that specifically targets TLR4-dependent signaling could also alleviate hyper-inflammation in the lungs [16]. In further support of this possibility, photobiomodulation therapy has been found to be effective in treating many inflammatory conditions including thyroiditis and psoriasis, which result from autoimmune hyper – inflammation [25–27,51,52]. Therefore, the effectiveness of light therapy as an anti-inflammatory treatment does not appear to be cell type specific or to occur only in isolated instances, and the results obtained in HEK cells likely being of general relevance. Finally, although there is not yet consensus, one proposed mechanism for red/infrared photobiomodulation treatment has been that stimulation of mitochondrial enzymes (for example, cytochromes), producing transient bursts of ROS (reactive oxygen species) [28,35]. ROS are implicated in regulating the immune response in complex ways; however, some reports on their effect on hyper-inflammatory responses are in keeping with a therapeutic role [35,53,54]

### 4.2 Practical considerations for use of infrared therapy to treat lung illness

Unlike most current applications of photobiomodulation therapy, treatment of lung inflammation requires deep, uniform penetration of light into the chest cavity. Unfortunately, many LED-based photobiomodulation devices on the market do not provide either the necessary intensity or a suitable wavelength for deep tissue penetration. Red light, for instance, does not effectively penetrate beneath the skin surface and generates high heat, as is true also of infrared heating lamps or incandescent bulbs, which emit at wavelengths above 900 nm. Exposure to many such lamps at the intensities necessary for chest therapy would result in burns to the skin, for example, an incandescent infrared light. Further practical considerations are with respect to the time-dependence of the therapy. Exposure times have to be quite precise, as even 5-min variation renders the treatment ineffective (Figure 2). However, we did not find a significant decrease in effectiveness even at much higher light intensities (Figs.3, 4), suggesting that too much light intensity is not a problem, and only the duration of exposure is critical. Thus, the main challenge is providing a uniform illumination at a high enough intensity to the patient’s thoracic cavity.

We have used a wavelength at 720–750 nm in our study as this provides penetration of tissue without engendering heat and has optimal effects on TLR4-dependent inflammation. As an approximation of light penetration, we have measured the passage of light through pork rib and 3 cm steak sections, and determined that a powerful enough LED to achieve a light intensity of at least 1000 W/m^2^ at the skin surface is required for light penetration of 1–2 W/m^2^ into the chest and lungs [36]. We have interchangeably used commercial high output 720 nm LED bulbs and floodlights in these studies (see methods), which do not unduly heat the skin surface (increase in 5°C after 10 m exposure) and also achieve an output intensity that would be sufficient to penetrate the chest cavity.

**Figure 6.**
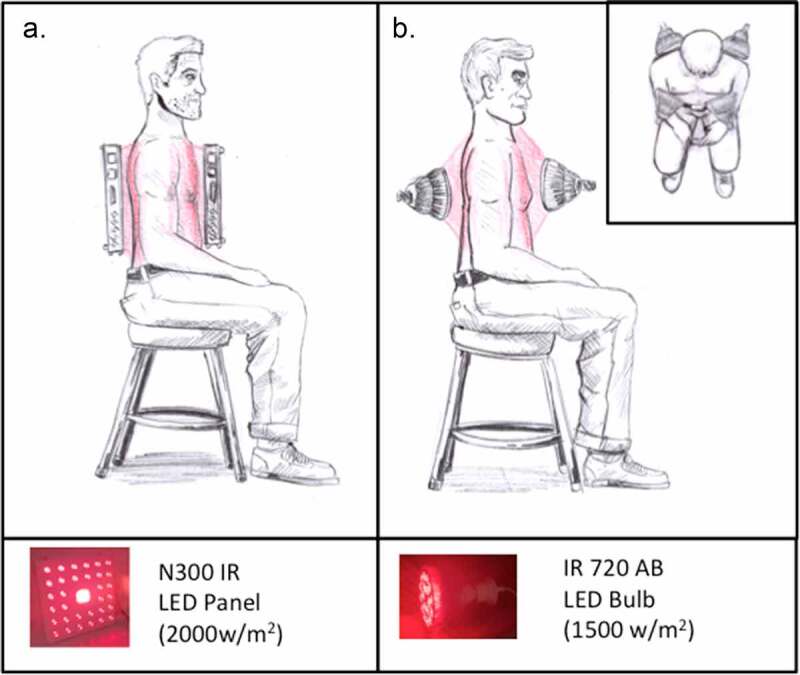
**Suggested application of infrared light sources to COVID-19 patients**. LED light sources of a suitable wavelength and light output intensity (minimum intensity of 1000 W/m^2^ at the skin surface) should be applied either: **A**. Floodlights (left panel) placed on either side of the patient or B. Four High Output LED Bulbs placed in front and behind patient’s chest area (right panel). lamps should be illuminated simultaneously to achieve maximum light penetration and timing precision

To achieve maximum effectiveness, a further consideration is the timing factor; all sections of the lung should be uniformly illuminated over a single optimal 10 m time interval. Therefore, light sources for an eventual treatment should be arranged to illuminate the entire chest surface as uniformly as possible, and from front and back simultaneously. This can be achieved, for instance, using four suitably positioned LED bulbs or two suitably positioned LED floodlights (Figure 6). Since most commercially available photobiomodulation devices do not fulfill the high output requirements, we caution against using them without first testing their wavelength and intensity to determine if they are suitable. The same considerations also apply to the use of photobiomodulation lasers.

### 4.3 Risk factors

Apart from the dangers of skin burns at a too-high exposure level, photobiomodulation therapy has no reported risks or risk factors, and has been approved for a range of medical applications [28]. Accordingly, it is not subject to the dangers of side effects inherent in drugs, such as tocilizumab and steroids, which are currently among the few agents being used effectively to treat late-stage respiratory distress [1,2,17]. On the contrary, a further major advantage of infrared therapy over drug-based treatments is that it can be targeted specifically to the organ or body part under attack. Drug-based anti-inflammatory treatments, which cannot be targeted to specific organs, will compromise the normal functioning of the immune system throughout the body, and thereby possibly inhibit the patient’s ability to fight off the viral infection. With photobiomodulation therapy, it should be possible to target infrared treatment exclusively to lung alveolar cells and resident macrophages in the chest area where the life-threatening damage is occurring, without affecting the immune response in other tissues.

## 5. Conclusion

As summarized in the Introduction, photobiomodulation therapy is a widespread treatment that has been used for a variety of conditions with underlying inflammatory issues for over 100 years. Furthermore, among the few anti-inflammatory drugs that are currently approved for late-stage COVID-19 infection, one (tocilizumab) is a drug used to suppress inflammation in rheumatoid arthritis, which is in fact a condition that also responds to photobiomodulation therapy [17,29]. Our results show that infrared exposure interacts with a key inflammatory response pathway that is deregulated in COVID-19 patients, as well as defining the critical exposure parameters. This treatment is affordable, simple to implement, has no known side effects, and is also extremely effective in our cell culture model system.

However, to prove the effectiveness of infrared therapy for treatment of COVID-19 will require performing controlled, clinical tests on patients in a hospital setting. In this respect, it is extremely encouraging that successful, small-scale reports exist of photobiomodulation therapy used for COVID-19 [37–39]. However, no large-scale clinical trial data are currently available, which will bring this therapeutic intervention into the mainstream. We hope that our results will help to provide a roadmap for which factors of wavelength, intensity, and most critically the time interval of light application are crucial so as to develop an optimal treatment protocol in patients.

Given the paucity of effective treatments for late-stage respiratory distress, we hope that our results will help to stimulate the necessary enthusiasm for taking this project to the next level and would be delighted to cooperate with any such endeavor.
